# Burn care: The challenges of research

**DOI:** 10.4103/2321-3868.123071

**Published:** 2013-12-18

**Authors:** Fiona M. Wood

**Affiliations:** 1 Burns Service of Western Australia; Burn Injury Research Unit, University of Western Australia, Australia; 2 Burn Injury Research Unit, School of Surgery, University of Western Australia, Crawly, Perth WA 6009 Australia

**Keywords:** Burn injury, translational research, improved outcomes

## Abstract

Burn care is an area which has advanced relentlessly over the past decades with improved survival and quality of survival. However, there are many challenges which overshadow the successes. The translation of what we know into clinical practice remains a challenge due to issues on many levels from overcoming personal opinion to resource allocation. We live in a time of exponential increase in knowledge in diverse areas which could be harnessed to improve the lives of those suffering burn injuries. Breaking down silos in education training and research remain challenging and again the allocation of resource is key. Ultimately when the goal is “One World One Standard of Burn Care” the greatest challenge is in education with specific reference to burn injury prevention and first aid.

## Introduction

Burn injury is associated with a complex pathophysiological response with rapidly cascading influences impacting on the whole of the patient with multisystem disruption.[1] The injury results in physical and psychological sequel such that every very intervention from the point of injury will influence the scar worn for life. The patient embarks upon a path which may be widely variable in terms of first aid, wound cleaning, prehospital care and preparation for transfer, pain management, resuscitation, surgery, wound care, nutrition, scar management, and functional and psychological rehabilitation.[2] Clearly the clinical problem faced on a daily basis is complex and research is essential in continuing to developing innovative solutions to solve the clinical problems.[3]

**Table 1:** 

Access this article online	
**Quick Response Code:**	**Website:** www.burnstrauma.com
	**DOI:** 10.4103/2321-3868.123071

Looking back at the accumulation of knowledge over the past decades demonstrates significant progress has been made with ongoing research being core to the development of burn care. With improvement in survival from burn injury the focus has shifted to the continuous improvement in the quality of survival.[4]

In order to give the patient the best evidence based treatment at every point in time from the time of injury, we need to consider three aspects;

1. The patient needs based on the clinical assessment at that point in time.
2. The experience/knowledge level of the treating clinician underpinned by ongoing education and training.
3. The environment of operation optimized based on prior knowledge.
  The concept of the “triangle of care” has been used to understand the capacity to treat the patient at a given time and drive the triage process.[5]

Bringing these elements together will dictate the outcome for that patient at that time guiding each intervention.[6] That is the starting point, bringing together what we know and delivering that knowledge to the bedside.[7]

## Challenges in the research of burn care

So where is the challenge?

- **Utilizing what we know effectively:** It has been stated that if current knowledge was implemented we would see a significant reduction in complications and concurrent improvement in outcomes. The translation of evidence into practice is an area in need of research and audit. The investigation of drivers and barriers to the implementation of evidence is vital to action timely translation into clinical practice. Pivotal to the continuous improvement in care is a systematic approach to education and training in the broadest sense. Community education ensuring appropriate use of first aid can have a significant impact on outcome.[8] There is no substitution for meticulous attention to detail; using what we know in the best capacity possible, then we can push the boundaries.[9]
- **Harnessing the potential opportunities from the ever expanding knowledge base in science and technology:** Pushing the boundaries, engaging in collaborations with basic science, population health, and clinical research to provide innovative solutions to complex clinical problems. Collaboration between disciplines provides real opportunities for improvements in clinical care, translating to improved outcomes for patients. We need to understand how the experimental situation, with control of all but one variable, can be extrapolated into the clinical situation. The design of clinical trials is an ongoing challenge due to the complex nature of the responses. A clear focus on burn injury is essential for a targeted problem solving approach and has facilitated great advances in care of the burn injured patient. However, this should not be at the expense of a broad general knowledge gaining insight into potential links and facilitating cross-fertilization.

Clinical practice is a fusion of experience and knowledge based on the observations of the natural history and the impact of interventions guiding advancements.

An essential element in observation is development of an appropriate measurement tool for each variable to facilitate the research process.[10] How do we measure the extent of the injury, the impact of the injury on the given individual, and the outcome post injury?

How do we measure and correct for events, both intrinsic and extrinsic to the patient, along such a complex pathway?

The initial measurement of the injury severity and scoring systems is vexed as complex factors are weighted to give scores to facilitate comparison.[11]

We have many examples linking wide ranging aspects of the individual demonstrating the complexity, such as the impact of premorbid personality on injury outcome the measurement of which is challenging and stimulates much debate.[12]

We also know that knowledge of the etiology is essential as we observe routinely the progression of a scald injury differing from flame injury. Understanding and documenting the detail in data collection is key to understanding the validity of comparison of either a single variable, or of a network of interacting variables.

Such tools as the laser Doppler scanner have the potential to assist in depth assessment, but it is influenced by many factors such as resuscitation and of course availability.[13] The use of laser body surface mapping has potential to percentage total body surface area, but relies on accurate definition of the edge of the injury and availability. The other known drivers to outcome such as age may be easily recordable but chronological age does not equate with physiological age in many individuals, with respect to concurrent pathologies and use of alcohol and drugs.[14]

Considering the measurement the impact of the multiple interventions post injury, attempts have been made to breakdown the measurement into aspects such as the domains of the Burn Specific Health Scale (BSHS) physical and psychological? Increasing efforts in the area of outcome measurement related to the burn injury have validated a combination of subjective and objective measures.

Progress has been made in the area of assessment and measurement, but is still a work in progress and an area of great potential as new technologies have the potential to shift the subjective to the objective. For example, the accurate reliable wound assessment and scar outcome is fundamental to clinical research and areas were many technologies have been explored.

- **Developing rigorous systems of validated measurement and data collection with transparent analysis published for the benefit of all the burn population:** As we develop and increasingly invest in robust research systems based on sound research governance, we have to understand the responsibility of research and in particular the publication of results with transparency. Steven E. Wolf, editor of BURNS has publicly stated on many occasions the responsibility to see the work through to the finish with publication is vital. The funding of research could be put forward as the greatest challenge for the future. Yes it is a challenge, but all of us engaged in the endeavor need to share by publication to be realistic regarding the worth of our work. Nothing should be wasted.[15]

The 50^th^ anniversary edition of the Medical Journal of Australia published the vision of clinical care in a number of disciplines in 50 years’ time. With respect to burn injury repair the aim is healing by regeneration and restoration of function. The vision was described;

“Assessment is key in understanding the extent of injury.

Debridement is focused on tissue salvage.

Reconstruction balances repair with regeneration.

Investigation of multimodality, multiscale characterization, including confocal microscopy and synchrotron technology will quantify assessment.

Debridement using autolytic inflammatory control techniques with image-guided physical methods will ensure the vital tissue frameworks are retained.

Tissue-guided regeneration afforded by self-assembly nanoparticles will provide the framework to guide cells to express the appropriate phenotype in reconstruction.

To solve the clinical problem a multidisciplinary scientific approach is needed to ensure the quality of the scar is worth the pain of survival.”

Within a decade many of the technologies highlighted are available and in need for research to move along the innovation pathway to ensure safe implementation into healthcare systems. Progress requires collaboration at all stages from basic science, clinical trial design to population health research with a link to health economics, driven by improved clinical outcomes.

We need to challenge our thinking and in particular revisit our preconceived ideas and “gold standards”. For example, we should set our sights higher than wound healing achieved by split thickness skin grafting which commits the patient to a lifelong scar. Rather, we should aim for regeneration of the skin to the preinjury state matched specifically for that body site.[16] There is an increasing understanding of variation in individual responses to therapeutic interventions; what is the role of *n* = 1 clinical trials?[17] Is such case reporting anecdotal or an opportunity to engage in individualized medicine, linking genetic knowledge with potential therapies to focus on the outcome for that single individual?

Understanding that every intervention from the time of injury influences the scar worn for life has driven research by the multidisciplinary burns teams in a multitude of directions; from first aid and prevention to stem cells to rehabilitation strategies to name a few.

We live in exciting times with tools which have the potential to identify novel therapeutic targets as we strive towards regenerative healing. The exploration of regeneration and the interplay between genes, cells and tissues is possible with advanced bioinformatics systems developing to understand the network interactions.[18]

There has been great progress in burn care driven by dedicated individuals focused on improving the outcome of burn patients over the past decades. The challenge we face now is to capitalize on that tradition and link with the opportunities afforded by the exciting and unprecedented growth in science and technology.[19]

As we drive to “One World One Standard of Burn Care” as championed by International Society for Burn Injuries (ISBI) at the 2012 international meeting, we need to understand the key drivers of outcome, standardize the care around the key elements, and then we may make progress towards meaningful comparisons.[20] The key to improvement in global outcomes is in the translation of research into practice. The essential element in the equation is the education at all levels from community injury prevention and first aid, to the dedicated burns team setting and all in between to ensure the quality of the outcome is worth the pain of survival.[21]
